# Field evaluation of the Bioline Malaria Ag P.f/Pan Rapid Diagnostic Test: Causes of Microscopy Discordance and Performance in Uganda

**DOI:** 10.21203/rs.3.rs-5629938/v1

**Published:** 2025-02-19

**Authors:** Kisakye Diana Kabbale, Bienvenu Nsengimaana, Francis D. Semakuba, Brian A. Kagurusi, Caroline Mwubaha, Innocent Wiringilimaana, Thomas Katairo, Shahiid Kiyaga, Monica Mbabazi, Samuel Gonahasa, Moses R. Kamya, Stephen Tukwasibwe, Sam L. Nsobya, Victor Asua, Daudi Jjingo, Bosco Agaba, Catherine Maiteki-Sebuguzi, Jimmy Opigo, Kylie Hilton, Sarah G. Staedke, Grant Dorsey, Melissa D. Conrad, Bryan Greenhouse, Isaac Ssewanyana, Jessica Briggs

**Affiliations:** Infectious Diseases Research Collaboration; Infectious Diseases Research Collaboration; Infectious Diseases Research Collaboration; Infectious Diseases Research Collaboration; Infectious Diseases Research Collaboration; Infectious Diseases Research Collaboration; Infectious Diseases Research Collaboration; Infectious Diseases Research Collaboration; Infectious Diseases Research Collaboration; Infectious Diseases Research Collaboration; Infectious Diseases Research Collaboration; Infectious Diseases Research Collaboration; Infectious Diseases Research Collaboration; Infectious Diseases Research Collaboration; African Center of Excellence in Bioinformatics and Data Intensive Sciences, Makerere University; Ministry of Health; Ministry of Health; Ministry of Health; University of California; Liverpool School of Tropical Medicine; University of California San Francisco; University of California San Francisco; University of California San Francisco; Central Public Health Laboratories; University of California San Francisco

**Keywords:** Malaria, Plasmodium falciparum, HRP2/pLDH combination Rapid Diagnostic Test, performance, specificity, sensitivity, discordance, pfhrp2, pfhrp3

## Abstract

**Background:**

Histidine Rich Protein 2 (HRP2)/pan-Lactate Dehydrogenase (pLDH) combination Rapid Diagnostic Tests (RDTs) may address the shortcomings of RDTs that detect HRP2 alone. However, the relative contribution of the possible causes of discordant results (RDT-negative and microscopy-positive) and performance in field settings are poorly quantified.

**Methods:**

This study utilized samples from two cross-sectional surveys conducted in 32 districts at 64 sites across Uganda between November 2021 and March 2023 that enrolled 6354 febrile participants ≥ two years of age. Discordant samples (negative by HRP2/pLDH RDT and positive by microscopy) underwent quantitative PCR (qPCR) to detect and quantify parasitemia. Those confirmed to be positive for *P. falciparum* at > 1 parasites/microliter (p/μL) were tested for *pfhrp2* and *pfhrp3* deletions using digital PCR. Those that were negative or had *P. falciparum* detected at ≤ 1 p/μL underwent *Plasmodium* species testing using nested PCR. The performance of the Bioline Malaria Ag P.f/Pan combination RDT was evaluated by comparison with microscopy and qPCR.

**Results:**

There were 166 (8.4%) discordant samples out of 1988 microscopy positive samples. Of these, 90/166 (54.2%) were confirmed to contain *P. falciparum* at levels > 1 p/μL whereas 76/166 (45.8%) were negative or had *P. falciparum* levels ≤ 1 p/μL. Only one *P. falciparum* positive sample was confirmed to have a deletion in *pfhrp3*. The primary reasons for RDT-negative, microscopy-positive discordance in samples testing negative for *P. falciparum* were non-falciparum species (37/76, 48.7%) or false positives by microscopy (31/76, 40.8%). The sensitivity of the Bioline Malaria Ag P.f/Pan combination RDT was high (> 91%) using either microscopy or qPCR as the gold standard. However, specificity was low (56.7%) when microscopy was used as the gold standard; it improved to 64.0% when qPCR was used as the gold standard.

**Conclusion:**

The Bioline Malaria Ag P.f/Pan combination RDT was found to be highly sensitive in Uganda and reliable for ruling out malaria. False negative RDT results were primarily due to low density *P. falciparum* infections, non-falciparum infections, or incorrect microscopy results. In contrast, false positive RDT results were common due to persistent antigenemia; this may result in overuse of antimalarial drugs and missed diagnoses of non-malarial febrile illnesses.

## BACKGROUND

In 2022, there were 249 million cases of malaria reported globally, and 95% of these were from the WHO African Region (1). Uganda is among the three countries with the highest burden of malaria, and 97% of the cases in the country are caused by *Plasmodium falciparum* (2). In addition, malaria is a leading cause of morbidity and mortality in Uganda, accounting for up to 50% of outpatient visits and up to 20% of inpatient admissions and deaths (3).

Parasitological confirmation of malaria by microscopy or rapid diagnostic tests (RDTs) is critical for effective case management and surveillance (1). Microscopy is the recommended gold standard for malaria diagnosis; however, high quality microscopy is time consuming and often unavailable in resource-limited settings. RDTs are a more feasible and scalable option because of their cost-effectiveness, ease of use and ability to provide quick results (4). Commercially available RDTs target three major antigens: HRP2, specific to *P. falciparum*, and *Plasmodium* lactate dehydrogenase (pLDH), and aldolase for identification of non-falciparum and mixed infections. RDTs that detect HRP2 cross-react with HRP3, which has an antigenic profile similar to HRP2; therefore, circulating HRP3 can trigger a positive result in the absence of HRP2 (5). In Uganda, RDTs that detect HRP2 are the recommended and preferred choice for malaria diagnosis because *P. falciparum* is the dominant species and HRP2-based RDTs have higher sensitivity and thermostability compared to those that detect pLDH (6). Furthermore, *P. falciparum* infections with double deletions of *pfhrp2/pfhrp3*, which render HRP2-based RDTs ineffective, are reported to be rare in Uganda (7, 8).

While the high sensitivity of RDTs that detect HRP2 is an advantage, persistent HRP2 antigenemia for several weeks after antimalarial treatment in high malaria transmission settings compromises the specificity of these RDTs for detecting clinical malaria (4). An advantage of HRP2/pLDH combination RDTs that detect both *P. falciparum* HRP2 and pLDH is that pLDH is cleared more quickly from the bloodstream after parasite clearance (9). Therefore, these tests may potentially reduce false positive results due to persistent HRP2 antigenemia if read as positive only if both HRP2 and pLDH bands are positive. Combination RDTs that include detection of pLDH have the additional benefit of detecting other *Plasmodium* species, which are also present in Uganda (10). Furthermore, modelling studies have shown a reduced risk of emergence of *pfhrp2/pfhrp3* deletions with the use of HRP2/pLDH combination RDTs compared to RDTs detecting HRP2 alone (11).

Because of their enhanced ability to distinguish clinical malaria from persistent antigenemia, the global threat of *pfhrp2/pfhrp3-deleted* parasites, increasing reports of non-falciparum *Plasmodium* infections in Uganda and enhanced thermostability, combination RDTs may become the preferred option for malaria diagnosis in the future. Understanding the causes of discordant findings, wherein microscopy is positive but combination RDTs are negative, will be important if a change is recommended to combination RDTs in the future. Therefore, a study was performed to examine the causes of discordant microscopy and HRP2/pLDH RDT combination results using dried blood spots (DBS) collected from febrile patients in cross-sectional surveys conducted in 32 districts at 64 sites across Uganda from November 2021 to March 2022 and November 2022 to March 2023. In these cross-sectional surveys, the Bioline Malaria Ag P.f/Pan combination RDT that detects both HRP2 and pLDH antigens was used. RDTs were read as positive if either the HRP2 or pLDH band was positive or if both bands were positive. The real-world performance of these combination RDTs when read in the field as positive using those criteria was also evaluated versus microscopy and quantitative PCR.

## METHODS

### Parent Study

**Figure 1:**
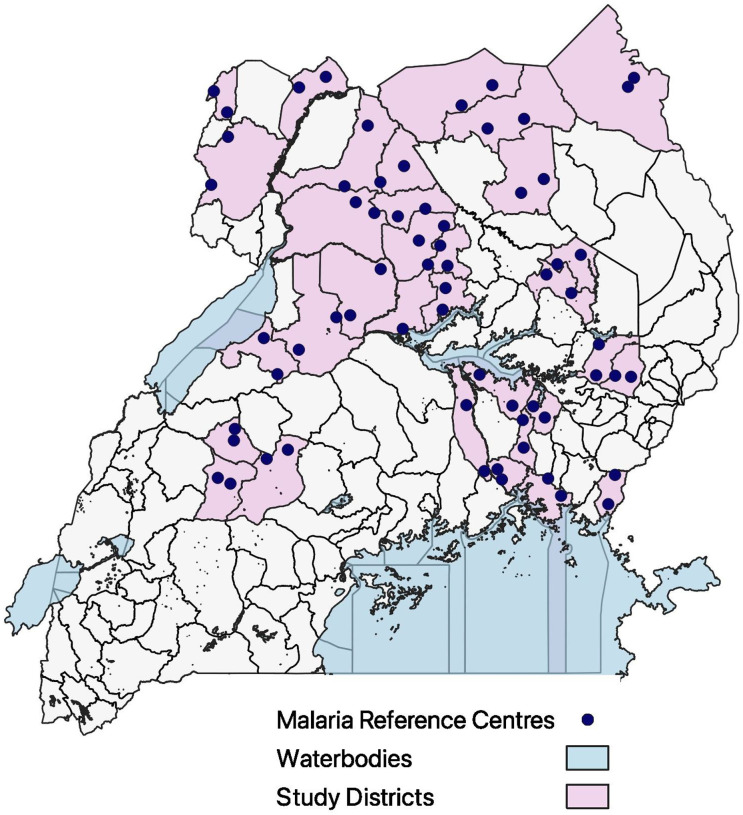
Map of Uganda showing the location of the 64 health facilities where cross-sectional surveys were conducted in the surrounding communities

This study was nested within the LLINEUP2 cluster randomized controlled trial of two types of long-lasting insecticide treated nets (LLINs); details of this study have been published elsewhere (12). Briefly, two cross-sectional surveys were conducted in the communities surrounding 64 health facilities in 32 districts at 12 and 24 months after the distribution of the nets to assess for parasite prevalence (Figure 1). The 12-month survey took place between November 2021 - March 2022, and the 24-month survey between November 2022 - March 2023. Fifty households with at least one child aged 2–10 years were enrolled at each site in both cross-sectional surveys.

In the 12-month cross-sectional survey, children ages 2–10 years were eligible for participation in all 64 sites; in 32 sites, adults were also eligible for participation. In the 24-month cross-sectional survey, only children aged 2–10 years were eligible for participation (12). Participants were enrolled if they were a resident of the household and present the night before the survey, they or their parent/guardian provided informed consent, and assent was provided for children 8–18 years of age. Data collected from all participants included measurement of temperature, subjective fever, and a finger-prick blood sample for preparation of thick blood smears and collection of a dried blood spot (DBS).

#### Rapid Diagnostic Tests

Any participant with a temperature of >= 38.0°C or who reported subjective fever in the past 48 hours had a rapid diagnostic test (RDT) performed using the Bioline Malaria Ag P.f/Pan, Abbott Diagnostics RDT which is WHO prequalified. RDTs were conducted according to the manufacturer’s instructions and reported positive if either the “Pf” or the “Pan” bands were positive or if both bands were positive. Participants with a positive RDT result were given antimalarial treatment following local guidelines.

#### Microscopy

Thick blood smears were dried and sent to the Infectious Diseases Research Collaboration Molecular Research Laboratory in Kampala. Slides were stained with 2% Giemsa for 30 minutes and read by experienced laboratory technologists. Parasite densities were calculated by counting the number of asexual parasites, per 200 leukocytes (or per 500, if the count was less than 10 parasites per 200 leukocytes), assuming a leukocyte count of 8000/μl. A thick blood smear was considered negative when the examination of 100 high power fields did not reveal asexual parasites. For quality control, all slides were read by a second microscopist and a third reviewer settled discrepant readings, defined as (1) positive versus a negative thick blood smear, (2) parasite density differing by >25%.

### Study Design

**Figure 2:**
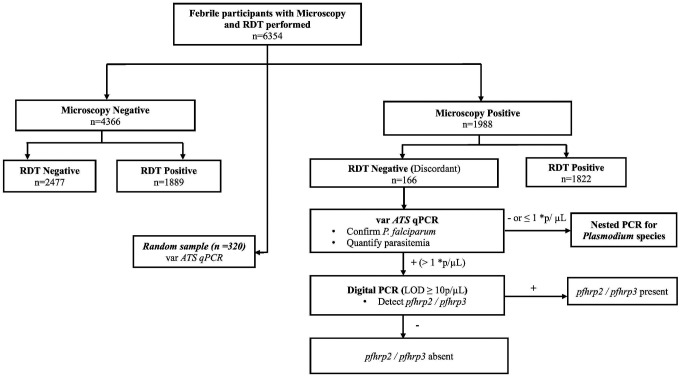
Sample testing workflow. Samples tested from the LLINEUP2 12- and 24-month surveys. RDT-negative/microscopy-positive samples (discordant samples) underwent testing to confirm the presence of *P. falciparum* by varATS qPCR, *pfhrp2/pfhrp3* deletions if positive, and non-falciparum infections if negative or low parasite density. 320 random samples were selected to calculate RDT performance metrics with qPCR as the gold standard. *p, parasites/μL

This study used DBS and microscopy results from participants enrolled in the cross-sectional surveys who consented to future use of biological specimens at the time of enrollment. Discordant samples were defined as RDT-negative and microscopy-positive. Sensitivity, specificity, negative predictive value (NPV) and positive predictive value (PPV) of the Bioline Malaria Ag P.f/Pancombination RDTs were calculated from all samples using microscopy as the gold standard. The same performance metrics were also calculated from a random sample (n=320) of the 12-month survey samples using *varATS* quantitative PCR (qPCR) as the gold standard (13). The workflow for the molecular testing of discordant samples is shown in Figure 2 and molecular assays are described in detail below.

### Laboratory Methods

#### Parasite DNA Extraction

DBS were stored at room temperature and shipped to the Uganda National Health Laboratory Services (UNHLS) and used for molecular testing of parasites. DNA was extracted from 6mm discs obtained from DBS using the Tween-Chelex-100 protocol as previously described (14).

#### Confirmation and quantification of *Plasmodium falciparum* DNA

The presence and quantity of *P. falciparum* DNA in discordant samples was established using a highly sensitive *varATS* qPCR for detecting *P. falciparum* (13). For this study, samples were considered positive for *P. falciparum* DNA if the parasite density was > 0.1 parasites/microliter (μL). Those that were positive at > 1 parasites/μL were tested for *pfhrp2/pfhrp3* deletions. Samples that were negative or with a parasitemia of < 1/μL were tested for non-falciparum species as shown in Figure 2.

#### Detection of non-falciparum species

The presence of non-falciparumspecies was determined using a ssrRNA nested PCR for *Plasmodium* species followed by gel electrophoresis as previously described (15).

#### Digital PCR to detect *pfhrp2* and *pfhrp3* deletions

A previously described digital PCR assay was used to screen samples for *pfhrp2* and *pfhrp3* deletions using the QIAcuity digital PCR System (16). The targets for this assay were *pfhrp2, pfhrp3* and *tRNA*, a single copy gene and internal control. Each gene/target was tagged with a distinct fluorophore. The reaction volume was partitioned into 8500 nanopartitions which were subjected to endpoint PCR, followed by quantification of the DNA template for each target. Samples with <1000 parasites/μL were run in duplicate, while those with ≥ 1000 parasites/μL were run in singlet. The number of amplified droplets containing DNA template (positive partitions) and containing no DNA template (negative partitions) for each target and sample were output by the QIAcuity Software Suite 2.2.0.26. For a sample to be analyzed for *pfhrp2* and *pfhrp3* deletions, > 1500 valid partitions were required per well and ≥ 5 partitions were required to be positive for the internal control *tRNA*. A sample was considered positive for *pfhrp2* or *pfhrp3*if ≥ 2 partitions were positive for the target and ≥ 5 partitions were positive for *tRNA*, and negative for *pfhrp2* or *pfhrp3*if < 2 partitions were positive for the target and ≥ 5 partitions were positive for *tRNA*. Using 3D7 DBS controls, the assay reliably detected *pfhrp2* and *pfhrp3* down to 10 parasites/μL. DD2 and HB3 controls diluted as low as 10 parasites/μL were used to verify that the assay was able to detect *pfhrp2* and *pfhrp3* deletions, respectively.

### Data analysis

Demographic information was extracted from the parent LLINEUP2 study databases. QGIS software was used to map study sites and the districts where the samples were collected (17). Data analysis was performed using the R statistical programming language, R version 4.3.2 (18). Age, gender, temperature, and parasite densitywere categorized and summarized as proportions. Among microscopy positive samples, characteristics were compared between concordant samples (RDT-positive) and discordant samples (RDT-negative). Comparisons of proportions were made using the Chi-squared test and comparison of median parasite densities were made using the Mann-Whitney U test. Performance metrics including sensitivity, specificity, PPV, NPV and the kappa statistic were calculated using the R package, epiR version 2.0.75 (19).

### Ethical Approval

This study was approved by the Makerere University School of Medicine Research and Ethics committee (2020-193), the Uganda National Council of Science and Technology (HS1097ES), University of California, San Francisco, Committee for Human Research (20-31769) and the London School of Hygiene and Tropical Medicine Ethics Committee (22615). This study only included samples from study participants who provided consent for future use of the samples that were collected during the cross-sectional surveys.

## RESULTS

Microscopy and RDT were performed on a total of 6354 symptomatic participants from the cross-sectional surveys (Figure 2). Of these, 1988 (31.3%) participants were positive for malaria parasites by microscopy. Of those who were positive by microscopy, 166 (8.4%) were negative by RDT (discordant). The samples with discordant results were further investigated to establish reasons for discordance, including *pfhrp2/3* deletions.

### Characteristics of participants with concordant and discordant RDT and microscopy results

Age and sex distribution of participants was similar in those with concordant and discordant RDT and microscopy results (Table 1). The majority of participants were 5 to 15 years old, and approximately half were male. A greater percentage of those with concordant results had a temperature of ≥ 38.0 °C, compared to those with discordant results (22.3% vs. 3.0%, p < 0.001). Only 29.3% (534/1822) of those with concordant results had a parasite density less than 1000 parasites/μL by microscopy, compared to 59.0% (98/166) of those with discordant results (p <0.001). Median parasite densities were higher in participants with concordant results compared to discordant results (3640 parasites/μL vs 600 parasites/μL, p < 0.001)

### Molecular analyses of discordant samples by varATS qPCR, nested species PCR and pfhrp2/pfhrp3 digital PCR

**Figure 3:**
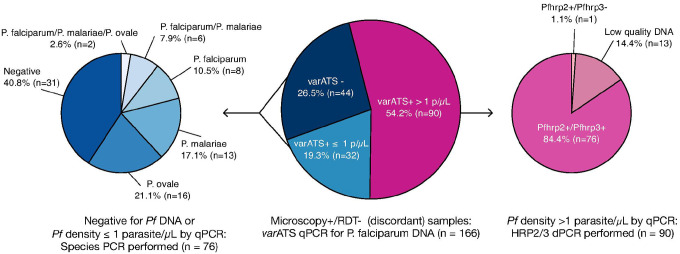
Molecular analyses of discordant samples by *varATS* qPCR, *pfhrp2/pfhrp3* digital PCR, and nested species PCR

The presence of *P. falciparum* at >1 parasites/μL was confirmed in 54.2% (90/166) discordant samples, while an additional 19.3% (32/166) were positive for *P. falciparum* at ≤ 1 parasites/μL by *varATS* qPCR (Figure 3). The remaining 26.5% (44/166) samples were negative for *P. falciparum* by *varATS* qPCR. Samples with parasitemia >1/μL underwent testing for *pfhrp2* and *pfhrp3* deletion using digital PCR (median parasite density, 242 parasites/μL). 14.4% (13/90) of these samples had fewer than 5 *tRNA* partitions and were excluded from further analysis due to low parasitemia (median parasite density was 5 parasites/μL). Of the 77 samples that passed the *tRNA* threshold, both *pfhrp2* and *pfhrp3* were detected in 98.7% (76/77) samples (median parasite density, 306 parasites/μL). Only one sample was found to have a deletion of *pfhrp3* (parasite density, 1,378 parasites/μL). There were no double deletions of *pfhrp2* and *pfhrp3* or single deletions of *pfhrp2* observed.

Seventy-six samples that were negative or positive at ≤ 1 parasites/μL by *varATS* qPCR underwent further testing by nested species PCR to determine if other *Plasmodium* species were present and might account for a discordant result with negative RDT and positive microscopy. Non-falciparum species and low-density falciparum infections were confirmed by nested species PCR in 48.7% (37/76) and 10.5% (8/76) of these samples, respectively (Figure 3). Mono-infections of *P. ovale* (21.1%, 16/76) and *P. malariae* (17.1%, 13/76) were the most common, followed by *P. falciparum* mono-infections (10.5%, 8/76) and mixed infections of *P. falciparum/P. malariae* (7.9%, 6/76) and *P. falciparum/P. malariae/P. ovale* (2.6%, 2/76). There were no *P. vivax* infections identified. The presence of non-falciparum species accounted for 22.3% (37/166) of the discordant samples. In 40.8% (31/76) of the samples that were negative or positive at ≤ 1 parasites/μL by *varATS* qPCR, no *Plasmodium* species could be identified by nested species PCR, implying that the microscopy result may have been a false positive. For samples that were negative by species PCR, expert microscopists re-read the slides, and the results were compared to the original field data. 30 of 31 slides originally read as positive were determined to be negative for *Plasmodium* species on re-read. One slide remained positive on re-read.

### Performance of the Bioline Malaria Ag P.f/Pan combination rapid diagnostic tests

Using microscopy as the gold standard, the sensitivity of the combination RDT in the LLINEUP2 study was high at 91.7% [95% CI 90.4 – 92.8] (Table 3). Specificity was relatively low at 56.7% [95% CI 55.2 – 58.2]. A negative test was highly accurate in predicting the absence of microscopic parasitemia, with a NPV of 93.7% [95% CI 92.7 – 94.6]. However, the probability of a positive test accurately predicting the presence of microscopic parasitemia, (PPV, 49.1% [95% CI 47.5 – 50.7]) was low. The level of agreement between the combination RDT and microscopy as measured by the kappa statistic was fair (k = 0.39, 95% CI 0.37 – 0.41).

Using varATS qPCR as the gold standard on a random sample of 12-month survey samples (n=320), the sensitivity of the Bioline Malaria Ag P.f/Pan combination RDT was 91.6% [95% CI 85.5 – 95.7], comparable to the sensitivity obtained using microscopy as the gold standard (Table 3). Specificity remained low at 64.0% [95% CI 56.7 – 70.9] but was higher than the specificity obtained when microscopy was used as the gold standard, due to RDT detecting some low-density infections identified using qPCR but not microscopy. This increment in specificity agreed with an increase in the kappa value to 0.52 [95% CI 0.43 – 0.61] for the Bioline Malaria Ag P.f/Pan combination RDT versus varATS qPCR. The NPV of the Bioline Malaria Ag P.f/Pan combination RDT remained high at 91.7% [95% CI 85.6 – 95.8] and the PPV improved to 63.8% [95% CI 56.5 – 70.7] when *varATS* qPCR was used as the gold standard.

## DISCUSSION

In this study, the relative contribution of the possible causes of discordant results (RDT-negative and microscopy-positive) and the performance of the Bioline Malaria Ag P.f/Pan combination RDT for malaria diagnosis in Uganda was evaluated using samples collected from symptomatic participants participating in 2 large cross-sectional surveys conducted at 64 different sites in 2021–2023. A low proportion (8.4%) of microscopy-positive samples were discordant. Patients with discordant results were less likely to have objective fever and had lower parasite density compared to patients with concordant results. The primary reasons for discordance were low density *P. falciparum* infections, non-falciparum infections, and false positive microscopy results. Discordant samples were assessed for *pfhrp2* and *pfhrp3* deletions by digital PCR. No *pfhrp2* deletions or double deletions were detected, and only one sample had a confirmed *pfhrp3* deletion. Consistent with these findings, HRP2/pLDH combination RDTs were found to be highly sensitive in this study. However, low specificity was observed regardless of the gold standard used (qPCR or microscopy), which is most likely due to the persistence of the HRP2 antigen after clearance of parasites in this high transmission setting where the majority of infections are caused by *P. falciparum* (2,7).

Discordant samples accounted for only 8.4% of the microscopy-positive samples. Among the discordant samples, most (54.2%) were low density *P. falciparum* infections detected by *varATS* qPCR (median parasite density of 241.9 parasites/μL). This is consistent with other studies that have shown that low density infections (<1000 parasites/μL) are associated with discordant RDT and microscopy results (8,20–24). Low density infections may not be detected by RDTs because they produce lower amounts of HRP2 and pLDH; most RDTs have a limit of detection (LOD) of 200 parasites/μL, under which the detection of HRP2 and pLDH is unreliable (25). Testing for *pfhrp2/pfhrp3* revealed that *pfhrp2* deletion or double deletions of *pfhrp2/pfhrp3* did not account for discordance in these samples. Even in the single sample with a *pfhrp3* deletion, *pfhrp2* was present, and parasite density was high enough to expect detection by RDT (1378 p/μL); the reason for RDT failure in this sample remains unclear and may have been caused by device or operator error.

Of the 76 discordant samples that were negative, or positive at less than 1 parasite/μL by *varATS* qPCR, 40.8% were negative by nested species PCR. These samples likely represent false positive microscopy results, which was confirmed for 30 out of 31 samples after re-examination by expert microscopists. False positive microscopy due to low quality microscopy in resource-limited settings has frequently been reported as a cause of RDT-negative/microscopy-positive discordance and is likely to be higher in real-world settings (21,24). Parr *et al*., 2021 reported a high proportion of false positives by microscopy (86%, 368/426) among discordant samples in the DRC (24). However, in the current study these represent 18.1% (30/166) of the discordant samples and only 1.5% (30/1,988) of the microscopy-positive samples. This is consistent with the low proportion of false positives by microscopy (10.9%, 24/219) among discordant samples reported by Agaba *et al*., 2020 in Uganda (21). The remainder of the discordant samples were positive by species PCR; of these, 82.2% (37/45) were positive for non-falciparumspecies or mixed infections. One study demonstrated poor sensitivities of 31.9% and 25% for the detection of *P. ovale* and *P. malariae* mono-infections respectively, by a pLDH based RDT (26). Non-falciparum mono-infections might, therefore, be missed by HRP2/pLDH combination RDTs. However, the prevalence of non-falciparum mono-infections is very low in Uganda, where 97% of the malaria infections are due to *P. falciparum* (2).

Because *pfhrp2/pfhrp3* deletions are a known cause of HRP2-RDT negative/microscopy positive discordance, discordant samples were screened for *pfhrp2/pfhrp3* deletions. In this study, the prevalence of *pfhrp2* and *pfhrp3* deletions cannot be directly estimated because the RDTs were read as positive if either antigen band or both antigen bands were positive and a *pfhrp2* deleted or double deleted parasite may have been pLDH positive. However, our findings are comparable to a 2024 study in Uganda that reported only one *pfhrp2* deletion using the WHO *pfhrp2/3* surveillance protocol to obtain samples from health facilities across Northern Uganda (7). Notably, in that study, only 50/2435 (2.1%) combination RDTs were HRP2-negative/pLDH-positive, and no deletions of *pfhrp2/pfhrp3* were identified in this subset. Therefore, a similar proportion of HRP2-negative/pLDH-positive RDTs would be expected in this study. Even if every one of these were caused by double deletions of *pfhrp2/pfhrp3* (which would be extremely unlikely), the prevalence of RDT and microscopy discordance caused by *pfhrp2/pfhrp3* deletions would not cross the WHO threshold of 5%. Based on the findings from this study and Agaba *et al*. 2024 (7), there remains no evidence that the prevalence of *pfhrp2/pfhrp3* deletions in Uganda exceeds the 5% threshold above which the WHO recommends a change in diagnostic policy (27). Older studies in Uganda have never reported prevalence of these deletions above this threshold (7,8,21,28,29). The low prevalence of RDT discordance due to *pfhrp2/pfhrp3* deletions in Uganda may be due in part to the high prevalence of polyclonal infections in high malaria transmission settings, which has also been reported in neighboring high malaria burden countries such as the DRC, Tanzania and Kenya (20,23,24,30). In polyclonal infections, a deletion of *pfhrp2* in one strain may be rescued by other strains in which *pfhrp2* is present; in these cases, the RDT will be positive (7,21,23,28,31). Furthermore, in parasites in which *pfhrp2* is deleted but *pfhrp3* is present, the HRP3 antigen may cross-react and produce a positive RDT result (4). While *pfhrp2/pfhrp3* deletions are not currently a threat to the use of RDTs detecting HRP2 in Uganda, it has been reported that their widespread use may drive clonal expansion of parasites with deletions of *pfhrp2* (32,33). One modelling study further demonstrated that the use of RDTs detecting HRP2 only selected for an increase in *pfhrp2* deleted parasites, while *P. falciparum* HRP2/pLDH combination RDTs did not (11). Therefore, HRP2/pLDH combination RDTs may become a preferred option for malaria diagnosis in Uganda in the future; however, their adoption would necessitate price reduction from $0.40 to match the $0.20 for HRP2-RDTs (34).

Molecular assays for the identification of *pfhrp2/pfhrp3* are challenging. Conventional PCR is time consuming, requires a high volume of DNA, and has diminished sensitivity at low parasite densities, while nested PCR is prone to contamination due to the multiple PCR steps required (35). Multiplex qPCR for *pfhrp2/pfhrp3* can be difficult to optimize for specific machines and settings (16,21,35). Attempts to optimize a multiplex qPCR assay for samples with parasite densities below 1000 parasites/μL were unsuccessful in this study (36). However, a dPCR assay that did not require extensive optimization was successfully used to identify *pfhrp2* and *pfhrp3* in the presence of *tRNA*, a single copy *P. falciparum* gene (16). The LOD for this assay was found to be 10 parasites/μL based on laboratory controls including DD2, D10, HB3, and 3D7; corresponding with this LOD, the median density of field samples without a reliable result was 5 parasites/μL. Therefore, this assay can confirm *pfhrp2/3* deletions in low density samples above a threshold of 10 parasites/μL.

In this study, the sensitivity of the Bioline Malaria Ag P.f/Pan combination RDT was found to be high at > 91%. Similarly, several studies have reported a high sensitivity of HRP2/pLDH RDTs at > 90% for the diagnosis of *P. falciparum* in high malaria transmission settings in DRC, Senegal, Ghana, Cameroon and Uganda (22,37–40). In addition, data from the current study show a high NPV of 91.7% - 93.7% for the combination RDT, which is consistent with that of RDTs detecting HRP2 in high transmission settings in Uganda (41,42) and suggests that HRP2/pLDH combination RDTs are highly accurate in ruling out malaria infection. The low specificity of the Bioline Malaria Ag P.f/Pan combination RDT in the current study (56.7%, which improved slightly to 64.0% when corrected by PCR) has previously been observed with RDTs detecting HRP2 (37,41,43–46). Higher specificity when PCR is used as the gold standard is expected because HRP2-based RDTs can sometimes detect submicroscopic infections that are also detected by qPCR(22,24,47). Murungi *et al*., 2017 also reported a low specificity of 46.7% in another study in Uganda where a HRP2/pLDH combination RDT was used to diagnose clinical malaria (39). This low specificity is likely due to the persistence of the HRP2 antigen in blood for several weeks after parasite clearance. One study in a hyperendemic region in Uganda reported persistent HRP2 antigenemia for a mean duration of 32 days, with a high pre-treatment parasitemia associated with a longer duration of persistence (42). Since HRP2 persists in blood and pLDH is cleared more rapidly, the specificity for the Bioline Malaria Ag P.f/Pan combination RDT may have been higher if the RDT result was considered positive only if both the pLDH and HRP2 bands were positive. Hawkes *et al*., 2014 and Boyce *et al*., 2017 demonstrated that the specificity of HRP2/pLDH combination RDTs for the diagnosis of clinical and severe *P. falciparum* malaria in high malaria transmission settings in Uganda improved from 62% to 82% and 52.1% to 89.1% respectively, when the RDT result was read as positive if both HRP2 and pLDH bands were positive (48,49). In the high malaria transmission setting of Uganda where HRP2-based RDTs are recommended, poor specificity of HRP2-only RDTs due to persistent HRP2 antigenemia likely results in inappropriate use of antimalarial drugs (49). It may also result into missed diagnoses of other non-malarial febrile illness. Thus HRP2/pLDH combination RDTs, if read properly, could potentially overcome the poor specificity of HRP2-based RDTs for the diagnosis of clinical malaria in high malaria transmission settings; however, there would be a compromise in the sensitivity of the test (49). Hawkes *et al*., 2014 reported a reduced sensitivity of 88% for HRP2-positive/pLDH-positive bands for the diagnosis of malaria among hospitalized children compared to 94% for HRP2-positive only (49).

The primary limitation of this study is that RDT positivity was reported regardless of whether the *P. falciparum* HRP2 or pLDH band was positive, and therefore, information was lost about how many RDTs were positive for HRP2, pLDH, or both. Though this is unlikely to significantly change the results, since the vast majority of malaria infections in Uganda are due to *P. falciparum* (2), this prevented assessment of sensitivity and specificity of the RDT if both lines were positive (HRP2-positive/pLDH-positive). In addition, due to study design, the prevalence of *pfhrp2* and *pfhrp3* deletions cannot be directly estimated (27). However, this study has a large sample size and good geographic representation across Uganda. Moreover, findings from the current study were concordant with the low prevalence of *pfhrp2/pfhrp3* deletions reported in a recent study in Uganda where samples were collected according to WHO guidelines (7).

In conclusion, false negative RDT results using the Bioline Malaria Ag P.f/Pan combination that detects both HRP2 and pLDH were uncommon. False negative results were typically due to low density *P. falciparum* infections, non-falciparum infections, or incorrect microscopy results. *Pfhrp2/pfhrp3* deletions remain rare in Uganda. The RDT demonstrated high sensitivity > 91% for the diagnosis of clinical malaria in the high transmission setting of Uganda and a high accuracy in ruling out malaria when read as positive if either or both bands were present. However, false positive results were common, likely due to persistence of HRP2 antigenemia, which may lead to overtreatment of malaria, misuse of antimalarial drugs and missed diagnoses of non-malarial febrile illnesses.

## Figures and Tables

**Table 1:** Characteristics of participants with concordant and discordant sample profiles

Characteristic	Concordant samples (Microscopy+ / RDT+)	Discordant samples (Microscopy+ / RDT−)
**Total** (n, %)	1,822	166
**Age in years** (n, %)		
<5	590 (32.4%)	60 (36.1%)
5 – 15	1172 (64.3%)	94 (56.6%)
≥16 years	60 (3.3%)	12 (7.2%)
**Male gender** (n, %)	949 (52.1%)	87 (52.4%)
**Temperature ≥ 38.0 °C** (n, %)	406 (22.3%)	5 (3.0%)
**Parasite density by microscopy**		
< 1000 parasites/μL (n, %)	534 (29.3%)	98 (59.0%)
Median parasite density in parasites/μL (Q1, Q3)	3640 (760, 12350)	600 (48, 2590)

**Table 3:** Performance of the Bioline Malaria Ag P.f/Pan combination rapid diagnostic tests using samples from LLINEUP2 surveys with microscopy and *varATS* qPCR as gold standards

Gold standard	Microscopy* (n = 6354)	qPCR^†^ (n=320)
	Value (95% CI)	Value (95% CI)
Sensitivity	91.7% (90.4 – 92.8)	91.6% (85.5 – 95.7)
Specificity	56.7% (55.2 – 58.2)	64.0% (56.7 – 70.9)
Positive Predictive Value	49.1% (47.5 – 50.7)	63.8% (56.5 – 70.7)
Negative Predictive Value	93.7% (92.7 – 94.6)	91.7% (85.6 – 95.8)

* True Positives (TP) = 1822, False Positives (FP)= 1889, True Negatives (TN) = 2477, False Negatives (FN) = 166

† True Positives (TP) = 120, False Positives (FP)= 68, True Negatives (TN) = 121, False Negatives (FN) = 11

## Data Availability

The code used to analyze the data from this study can be found at: https://github.com/dkisakye/Rfalciparum_HRP2_3_project.git. The LLINEUP2 datasets are available in the study database and will be publicly accessible upon publication.

## Abbreviations

P. falciparum: Plasmodium falciparum
Pfhrp2: Plasmodium falciparum histidine rich protein 2 gene
Pfhrp3: Plasmodium falciparum histidine rich protein 3 gene
HRP2: Histidine Rich Protein 2
HRP3: Histidine Rich Protein 3
DNA: Deoxyribonucleic Acid
varATS: var-Acidic Terminal Segment gene
tRNA: Transfer Ribonucleic Acid gene
RDT: Rapid Diagnostic Test
qPCR: Quantitative Polymerase Chain Reaction
pLDH: *Plasmodium* Lactate Dehydrogenase
LLIN: Long Lasting Insecticide-treated nets
DBS: Dried Blood Spot
dPCR: Digital Polymerase Chain Reaction
PPV: Positive Predictive Value
NPV: Negative Predictive Value
DRC: Democratic Republic of Congo
